# Implementation strategies, outcomes and determinants of integrated care for cardiometabolic conditions and mental, neurological and substance use disorders: protocol for a mixed-methods systematic review

**DOI:** 10.1136/bmjopen-2026-125828

**Published:** 2026-10-21

**Authors:** Emma Jeltje Blinker, Gordon Kwasi Ampomah Amoh, Samuel Ankamah, Charles Agyemang, Bernard Omech, Erik Beune, Frederick Murunga Wekesah, Marilyn Azumah, Wouter Munneke

**Affiliations:** 1 Department of Public and Occupational Health, Amsterdam UMC Locatie AMC, Amsterdam, Netherlands; 2 Emmanuel Quaye Archampong Library, University of Ghana, Accra, Ghana; 3 Graduate Training and Research Centre, Lira University, Lira, Uganda; 4 Health and Systems for Health, African Population and Health Research Center, Nairobi, Kenya; 5 Julius Global Health, University Medical Centre Utrecht, Utrecht, Netherlands; 6 Centre for Global Epilepsy, University of Oxford, Oxford, UK

**Keywords:** Implementation Science, Noncommunicable Diseases, Delivery of Health Care, Integrated, HEALTH SERVICES ADMINISTRATION & MANAGEMENT, MENTAL HEALTH

## Abstract

**Abstract:**

**Ιntroduction:**

The co-occurrence of cardiometabolic diseases and mental, neurological and substance use disorders health conditions represents a growing global health burden, particularly in low- and middle-income countries. Although integrated, people-centred multimorbidity care is increasingly recognised as essential across settings, evidence remained limited on how these models are operationalised in routine practice and which implementation strategies are most effective to support their delivery. This protocol outlines the methods for a systematic review aiming to identify implementation strategies used to integrate care for cardiometabolic conditions and mental, neurological and substance use (MNS) disorders, examine associated implementation outcomes and explore contextual determinants influencing implementation across settings.

**Methods and analysis:**

This protocol follows the Preferred Reporting Items for Systematic Review and Meta-Analysis Protocols (PRISMA-P) guidelines. A comprehensive search will be conducted in PubMed, CINAHL, APA PsycINFO, Scopus and Web of Science and relevant grey literature sources. Eligible studies include randomised controlled trials, quasi-experimental studies, mixed-methods studies, qualitative studies and implementation evaluations reporting on integrated care models addressing both cardiometabolic conditions and MNS disorders. To be included in this Systematic Review, studies must detail strategies or models integrating care for at least one cardiometabolic condition and one MNS disorder and provide data on implementation outcomes or contextual determinants. Title/abstract screening, full-text screening, data extraction and data synthesis will be conducted independently by at least two researchers following pilot testing. A formal risk of bias assessment will be performed using the Mixed Methods Appraisal Tool. A mixed-method, framework-based synthesis will be undertaken. Implementation strategies will be coded using the Expert Recommendations for Implementing Change taxonomy, implementation outcomes using Proctor *et al*’s taxonomy, and contextual determinants using the Consolidated Framework for Implementation Research. Quantitative data will be summarised descriptively using frequencies, proportions and summary tables, with findings stratified by country income level, setting and type of integrated care model where possible. Qualitative data will be analysed using inductive coding and deductive mapping to implementation science frameworks.

**Ethics and dissemination:**

Ethical approval is not required as no primary data will be collected. The findings of this study will be disseminated through peer-reviewed publications, conference presentations, and stakeholders’ engagement in the field.

**Registration details:**

The protocol was registered with the International Prospective Register of Systematic Reviews (PROSPERO) on 6 April 2026 (registration number CRD420261321825).

**STRENGTHS AND LIMITATIONS OF THIS STUDY**

- This systematic review protocol is the first to use a mixed-methods approach to synthesise evidence on implementation strategies, outcomes and determinants of integrated cardiometabolic and mental, neurological and substance healthcare.
- In accordance with Preferred Reporting Items for Systematic Review and Meta-Analysis Protocols (PRISMA-P), rigorous methods are described on conducting systematic reviews, including the librarian-supported literature search, inclusion criteria, dual screening and reporting.
- This study will search journals from interdisciplinary fields, grey literature and forward- and backward citation searching in order to minimise the risk of missing relevant studies.
- The protocol outlines a structured approach for integrating quantitative and qualitative evidence using established implementation science frameworks (Expert Recommendations for Implementing Change taxonomy, Proctor outcomes and Consolidated Framework for Implementation Research).
- High anticipated heterogeneity in study methods, implementation strategies and outcome definitions limits how precisely synthesis procedures can be pre-specified in the protocol.

## Introduction

 Non-communicable diseases (NCDs), particularly cardiometabolic conditions such as cardiovascular disease, represent a rapidly growing global health burden. This burden is especially pronounced in low- and middle-income countries (LMICs), where 80–85% of NCD-related deaths occur, and nearly half of these deaths are premature.^1–6^Mental, neurological and substance use (MNS) disorders are likewise major contributors to global disability and leading cause of years lived with disability. They frequently co-occur with cardiometabolic diseases due to shared biological, behavioural and social pathways, leading to a bilateral causality.^7–11^

Their co-occurrence results in complex multimorbidity associated with poorer clinical outcomes (e.g. suboptimal blood pressure and glycaemic control, depression symptoms**,** poor quality of life), reduced treatment adherence, higher service use and costs and increased mortality compared with single conditions.^12–15^Addressing this complex multimorbidity requires coordinated, people-centred care, yet most health systems remain organised around single-disease programmes, limiting access to coordinated people-centred care and contributing to suboptimal quality of care.^3 16–20^ This is defined as integrated care by one of the working definitions ‘Initiatives seeking to improve outcomes for those with (complex) chronic health problems and needs by overcoming issues of fragmentation through linkage or coordination of services of different providers along the continuum of care’.^21^

Evidence from high-income countries (HIC) suggests that integrated care, specifically collaborative care and stepped care models can improve clinical outcomes (eg, glycaemic control), patient experience and cost effectiveness, although effectiveness varies depending on context and implementation fidelity.^22–28^ In addition, clinical guidelines increasingly acknowledge the interaction between MNS disorders and cardiometabolic conditions, recommending symptom screening, supporting medication adherence and applying stress-reducing interventions to improve clinical outcomes.^29–32^ The American Diabetes Association Standards of Care advises routine psychosocial assessment and collaborative, interprofessional diabetes care teams.^33^ National Institute for Health and Care Excellence (NICE) guidelines similarly call for integrated mental and physical healthcare, emphasising comorbidity management and collaborative care for patients with depression and chronic physical illness.^34 35^However, most clinical guidelines provide limited operational guidance. The WHO Package of Essential Noncommunicable Disease Interventions (PEN) and mental Health Gap Action Programme (mhGAP) offer condition-specific guidance for cardiometabolic conditions and MNS disorders in primary care delivery in LMIC but provide little direction on integrating services in routine practice.^7 36^

To effectively guide implementation and operationalisation, it is necessary to understand which implementation strategies are appropriate, implementable, what implementation outcomes they achieve, and understand the contextual determinants that explain these outcomes, alongside considerations for scale-up. However, existing reviews have primarily examined the effectiveness of integrated care models^37–41^or described the needs, barriers, and facilitators related to integrated care, such as environmental context and resources, and social influences.^42–45^Yet, these studies do not report strategies to address these barriers and facilitators or assess whether such strategies were successful. The scoping review of Adler *et al* identified a wide variety of strategies for integrated care, including frequent implementation of health education, screening, linkage to care, initial diagnosis, and medical dispensing, and patient follow-up and monitoring.^40^ However, this review only focused on LMIC and it remains uncertain if these strategies were implemented successfully or addressed context-specific barriers, particularly in LMICs, where fragmented financing, parallel reporting systems, weak referral networks, limited infrastructure and workforce shortages may shape whether integrated care can be adopted, delivered with fidelity and sustained over time.^17 19 20 46 47^It is also unknown whether this variation reflects contextual differences or gaps in understanding which implementation strategies are successful. At the same time, evidence from other areas of integrated service delivery, such as multimorbidity management within HIV programmes, demonstrates that integration can be feasible in low-resource settings when adapted to local delivery systems.^19 48–55^

Therefore, a systematic examination of implementation of integration of cardiometabolic and MNS healthcare from a global perspective is needed to identify implementation strategies, assess their successes or challenges, and to inform future studies aimed at strengthening multimorbidity care. By comparing how implementation varies across LMIC and HIC and stratifying findings by income level, we can distinguish strategies that are transferable from those that are context-dependent. Without this perspective, strategies may be transferred without necessary adaptation, or context-specific innovations may remain invisible in the global evidence base.^56 57^

Therefore, the primary aim is to narratively synthesise the implementation strategies used to integrate care for cardiometabolic diseases and MNS disorders across global settings, and their reported implementation outcomes.

Secondary aims are:

1. To identify which implementation strategies are commonly applied to integrate care for cardiometabolic diseases and MNS disorders.
2. To identify the frequency of implementation outcomes (eg, acceptability, feasibility, fidelity, adoption, sustainability and cost) examined in relation to integrated care.
3. To identify which determinants (eg, health system capacity, workforce, cultural context and financing) influenced successful implementation of integrated care.
4. To identify how implementation strategies and reported outcomes differ between LMICs and HICs.

## Μethods

This protocol describes an implementation-focused mixed-methods systematic review, allowing for broader evidence inclusion and supporting the use of implementation science frameworks. The protocol was registered with the International Prospective Register of Systematic Reviews (PROSPERO) on 6 April 2026 (registration number CRD420261321825).^58^

The protocol has been developed in accordance with the Preferred Reporting Items for Systematic Review and Meta-Analysis Protocols (PRISMA-P) guidelines.^59^

### Eligibility criteria

Eligibility criteria will be defined using the Population, Intervention, Comparators and Outcomes (PICO) framework. Studies will be included if they meet the PICO criteria, see table 1. The criteria are outlined below.

**Table 1:** PICO criteria for included studies

Population	All healthcare settings, health systems, community-based services and faith-based organisations. This can include but is not limited to: patients, healthcare providers and policymakers.
Ιntervention	Integrated care models combining or aiming to combine care for cardiometabolic conditions and MNS disorders. This can include current models and implementation strategies to integrate care.
Comparator	No comparator is required.
Outcomes	Quantitative and qualitative data regarding implementation outcomes (eg, acceptability, adoption, appropriateness, feasibility, fidelity, sustainability, penetration and costs). Studies reporting barriers, facilitators or stakeholder experiences will also be included.
Study type	Randomised controlled trials, quasi-experimental studies, hybrid effectiveness-implementation trials, process or implementation evaluations, mixed-methods studies, implementation reports and qualitative research describing implementation processes and outcomes.
Language	All languages.

MNS, mental, neurological and substance use; PICO, Population, Intervention, Comparators and Outcomes.

### Inclusion and exclusion criteria

#### Study designs

Eligible study types will comprise randomised controlled trials, quasi-experimental studies, process or implementation evaluations, mixed-methods studies, implementation reports, and qualitative research describing implementation processes, this includes but is not limited to in-depth interviews, key-informant interviews, focus-group discussions, ethnographic studies, case studies, and participatory or action research. Case series, case reports, abstracts, reviews, and protocols and studies without original empirical data, including letters, commentaries, editorials, will be excluded.

#### Population

Studies will be included if they examine health systems, healthcare organisations, primary care settings, community-based services, or faith-based organisations. This includes, but is not limited to, patient populations, healthcare providers, managers, and policymakers.

#### Interventions

Studies should describe either interventions for, or the implementation of, integrated care models that combine cardiometabolic and MNS management through linkage or coordination of services within a shared care pathway for adults. Integration can include, but is not limited to patient, provider or system level and include co-delivery of services, task-sharing, collaborative care, stepped care, shared clinical protocols, digital integration, multidisciplinary teams or a single provider managing both conditions. Interventions will be excluded if they focus on a single condition, parallel uncoordinated services, or referral only models without shared management.

Cardiometabolic conditions are defined using WHO-PEN, including type two diabetes and cardiovascular diseases including hypertension, myocardial infarction, stroke, heart failure and peripheral vascular disease.

MNS disorders are defined in accordance with the WHO mhGAP, including depression, anxiety, post-traumatic stress, dementia, epilepsy and seizures, psychosis and bipolar disorder, and self-harm and suicide. Studies focusing on child and adolescent MNS disorders will be excluded, as these were outside the scope of this review.

#### Outcomes

Eligible studies should report implementation-related outcomes for integrated care delivery (eg, acceptability, adoption, appropriateness, feasibility, fidelity, sustainability, penetration and costs). Studies that do not report these outcomes but do report implementation determinants (eg, barriers and facilitators) or stakeholder experiences with integrated care will also be included. Studies reporting only clinical effectiveness or clinical outcomes, without any implementation outcomes, determinants or stakeholder perspectives, will be excluded.

#### Setting

Any healthcare setting in any income level is eligible.

#### Language

Studies published in any language will be eligible. For titles and abstracts in languages not spoken by the review team, AI-machine translation will be used during screening. Full texts of potentially eligible studies will be translated using machine translation and, where feasible, checked with support from fluent speakers. The number and languages of non-English studies included and translated will be reported.

### Search strategy

A comprehensive search strategy is developed in consultation with an information specialist. Database searches are conducted in PubMed, CINAHL, APA PsycINFO, Scopus and Web of Science, combining MeSH terms and free-text keywords related to cardiometabolic diseases, MNS conditions, integrated care, implementation strategies and implementation outcomes including determinants, barriers, facilitators and stakeholder experiences. Search strings are adapted to each database to maximise retrieval sensitivity. Full search string for PubMed database is provided in online supplemental material. Grey literature is searched via ClinicalTrials.gov, Global Health, African Index Medicus, WHO IRIS, WHO NCD Document Repository, Ministry of health reports, The Cochrane Library, African Journals Online. Reference lists of included studies and relevant reviews are manually screened, and additional studies identified through forward- and backward citation searching. No date or language restrictions are applied.

### Data management

All identified records will be imported into Covidence, duplicates will be removed, where screening and data extraction will be managed. Pilot screening exercise will be conducted in Rayyan to calibrate eligibility criteria prior to full screening.^60^

### Selection process

Study selection will be conducted in two stages: title and abstract screening and full-text screening.

Prior to full screening, a pilot screening of a random sample (eg, 5%) of PubMed records will be conducted in Rayyan by all reviewers to ensure consistency and agreement of inclusion. Any discrepancies identified during the pilot will be discussed and resolved. All records will be independently double screened against the eligibility criteria using Covidence. Any discrepancies will be resolved through a final vote of a third reviewer after discussion. The study selection process will be reported using a PRISMA flow diagram. Non-English studies will be translated using AI tools (eg, DeepL) or native-speaking collaborators. Native speakers will verify translations when available; otherwise, AI-generated translations will be used.

### Data collection process

A standardised data extraction form will be developed in line with PRISMA recommendations and piloted on approximately 10% of included studies then refined. Each study will receive two independent data extractions, with discrepancies resolved through discussion and involvement of a third reviewer if necessary. Study authors will be contacted to obtain or clarify missing data; all decisions regarding missing data will be transparently documented. Data collection is planned to take place from August 2026 to January 2027.

#### Quantitative data extraction

The following items will be extracted numerically or categorically: study characteristics (design, sample size, setting, country income level, duration), provider characteristics (cadre, training, supervision structure), integrated care model type (condition combination, care components), and reported implementation outcomes (acceptability, feasibility, fidelity, adoption, appropriateness, penetration, sustainability, cost). Implementation strategies described in each study will be coded deductively using the Expert Recommendations for Implementing Change (ERIC) taxonomy. Contextual determinants reported quantitatively (eg, survey items on barriers or facilitators) will be extracted as presented. All categorical data will be extracted alongside their reported frequencies or proportions.

#### Qualitative data extraction

Full text or verbatim excerpts describing implementation strategies, contextual barriers and facilitators, stakeholder experiences and qualitative reflections on implementation outcomes will be extracted in their original phrasing. Where studies report mixed methods, the qualitative component will be extracted separately. Non-English qualitative data will be handled according to the translation approach described above.

### Risk of bias in individual studies

Methodological quality will be assessed using the Mixed Methods Appraisal Tool (MMAT), V.2018, which is suitable for qualitative, quantitative and mixed-methods studies.^61^ Separate design-specific risk-of-bias tools will not be applied because the review focuses on implementation strategies, outcomes and determinants rather than intervention effectiveness.

### Data synthesis

To address the review questions, the synthesis will identify patterns in how integrated-care models, implementation strategies, and outcomes are co-reported across studies. We will conduct a mixed-methods, framework-based convergent segregated synthesis following the Joanna Briggs Institute (JBI) methodology for mixed-methods systematic reviews.^62–64^In this design, quantitative and qualitative data are analysed independently, using methods appropriate to each data type, and subsequently integrated at the level of findings, followed by integration of the two sets of findings. For mixed-methods studies, quantitative and qualitative components will be extracted separately, ensuring that both contribute to the synthesis. Quantitative data will be synthesised narratively, given the heterogeneity of outcomes and the absence of effect sizes, while qualitative data will be analysed inductively through thematic approaches. During integration of both findings, quantitative and qualitative evidence will be given equal priority. This data synthesis identifies narrative patterns and does not imply statistical or causal relationships.

#### Quantitative analysis

Quantitative data will comprise the reported numerical or categorical findings from included studies: proportion of studies reporting each implementation strategy, the frequency of each implementation outcome as defined by Proctor *et al*’s taxonomy (acceptability, appropriateness, feasibility, fidelity, adoption, penetration, sustainability, cost) and the prevalence of reported contextual determinants.^65^

These data will be summarised descriptively using frequencies and proportions, presented in tables disaggregated by country income level, setting (primary, secondary, community) and type of integrated care model (eg, diabetes-depression vs hypertension-anxiety). No secondary statistical analysis or effect-size pooling will be performed; the quantitative synthesis remains descriptive.

#### Qualitative analysis

Qualitative data will comprise descriptive information about integrated care models, implementation strategies, verbatim excerpts, stakeholder narratives, case descriptions and open-ended survey responses from included studies. These will first be analysed inductively through open coding to identify emergent characteristics and themes. To ensure conceptual clarity between a model of care and implementation strategies, we define a model of care, in line with WHO, as the overarching concept for how services are delivered, including the processes of care, the organisation of providers and the management of services.^66^ The key characteristics of each integrated-care model will be synthesised and reported in a structured table. Implementation strategies are defined, following Proctor *et al*, as ‘methods or techniques used to enhance the adoption, implementation, and sustainability of a clinical program or practice’.^67^ Each strategy will be documented with its name, conceptual definition and operationalisation. The inductive codes of implementation strategies, outcomes and determinants will then be deductively mapped onto three distinct implementation framework components:

1. Implementation strategies: will be coded to the ERIC taxonomy using the ERIC matching tool.^68^
2. Implementation outcomes will be coded to Proctor *et al*’s taxonomy.^65^
3. Contextual determinants (barriers and facilitators) will be coded to CFIR domains: intervention characteristics, outer setting, inner setting, characteristics of individuals and process.^69^

Data will be independently coded by two reviewers, with discrepancies resolved through discussion and involvement of a third reviewer if necessary.

#### Integration of quantitative and qualitative findings

Following the independent syntheses, findings will be integrated using a narrative synthesis approach consistent with the JBI convergent segregated methodology.^62–64^ In practice, this means that quantitative findings (eg, ‘80% of LMIC studies reported low fidelity’) and qualitative findings (eg, ‘staff described inadequate supervision as a reason for low fidelity’) will be juxtaposed per implementation construct to identify convergence, complementarity or dissonance.

A narrative synthesis will be produced by organising findings around models of care and each of the three framework components (strategies, outcomes, determinants), with quantitative patterns and qualitative themes presented side by side. The synthesis will describe what the quantitative data show (how often, in which settings), what the qualitative data add (how and why things work or do not work), and where the two strands agree, diverge or highlight gaps. Cross-study comparisons will explore patterns across settings, health system levels and country income groups. Cost data, where reported, will be summarised narratively alongside the synthesis to clarify resource implications.

#### Subgroup analysis

Where data allow, subgroup analyses will be pre-specified to explore heterogeneity across four dimensions. For each dimension, both quantitative and qualitative data will be examined to identify patterns within subgroups (eg, within LMICs only or within primary care only) and to compare patterns between subgroups. Primary subgroups will include country income level and health system level/setting, as these are the most policy-relevant dimensions for implementation in LMICs. Exploratory subgroups will be type of integrated care model and type of implementation strategy, which may have fewer studies per category. Analyses for exploratory subgroups will be conducted only when at least three studies are available per subgroup category; where this threshold is not met, findings will be described narratively and limitations noted.

1. Type of integrated care model (exploratory)

- Quantitative: to compare the frequency and distribution of reported implementation strategies (ERIC), implementation outcomes (Proctor) and determinants (CFIR) across model types, such as diabetes–depression models versus hypertension–depression versus multi-condition models.
- Qualitative: to compare emergent themes and coded CFIR determinants across model types to assess whether barriers and facilitators differ by condition combination.

1. Type of implementation strategy (exploratory).

- Quantitative to compare the reported implementation outcomes (Proctor) between studies using different ERIC strategy clusters (eg, training and supervision vs technical assistance vs audit and feedback).
- Qualitative: to compare how stakeholders describe the experience, feasibility or perceived effectiveness of different strategy types, identifying qualitative patterns in what makes a strategy succeed or fail in context.

1. Health system level and setting (primary)

- Quantitative: to compare frequencies of strategies, outcomes and determinants across community-based versus facility-based care, primary versus secondary/tertiary care and urban versus rural contexts.
- Qualitative to compare coded barriers and facilitators by setting, exploring whether determinants such as infrastructure, workforce availability or referral pathways are reported differently across levels of care.

1. Country income level (primary)

- Quantitative: to compare the distribution of implementation strategies, outcomes and determinants between LMICs and HICs, and where possible distinguish lower-income from middle-income settings (eg, proportion of studies reporting task-shifting as a strategy in LMICs vs HICs).
- Qualitative: to compare themes and coded CFIR determinants across income groups to assess whether contextual factors (eg, financing constraints, workforce shortages, leadership support) manifest differently depending on country income level.

Together, these subgroup analyses will support the interpretation of heterogeneity and help identify context-specific patterns in implementation.

### Patient and public involvement

This review does not plan to involve patients or the public in the planning or conduct of the research.

### Study quality

Study quality will be assessed using MMAT, V.2018.^61^

### Certainty assessment

Formal certainty assessment will not be conducted in this review. The quantitative synthesis is descriptive and does not produce pooled estimates or effect sizes. For the qualitative component, certainty of findings will be assessed narratively through MMAT, transparency of coding procedures, and framework-based categorisation, rather than through a formal tool. This decision reflects the exploratory nature of the review and the heterogeneity of included study designs and settings, which limits the applicability of standardised certainty frameworks.

### Meta-bias

No formal assessment of publication bias or other meta-biases will be conducted.

### Confidence in cumulative evidence

The overall certainty of the evidence will not be formally assessed using Grading of Recommendations, Assessment, Development, and Evaluation (GRADE) or other frameworks, as the focus of this review will be a descriptive synthesis.

## Discussion

This will be the first mixed-methods systematic review to synthesise and report implementation strategies for integrating cardiometabolic and MNS healthcare with their associated implementation outcomes, determinants, barriers and facilitators.

Its strengths are the methodological contribution in using three validated implementation science frameworks: ERIC^68^ to classify implementation strategies, Proctor outcomes^65^ to structure implementation outcomes and CFIR^69^ to capture contextual determinants. The mixed-methods design strengthens the explanatory power of the review by integrating quantitative patterns (what was implemented, how often, and with what outcomes) with qualitative insights (why implementation succeeded or failed). The broad inclusion of study designs, settings (from community-based to tertiary care) and both LMIC and HIC contexts will further enhance the relevance of the findings for diverse health systems. Inclusion of grey literature and the absence of language restrictions further reduce the risk of language and publication bias, which is particularly important in implementation research.

Several methodological limitations must be acknowledged. First, although methodological quality will be assessed using the MMAT V.2018,^61^ no separate design-specific risk-of-bias tools will be applied. This decision is appropriate for a review focused on implementation strategies and outcomes rather than evaluating intervention effectiveness. However, it may constrain comparability across studies.^68 70^ Formal certainty assessment will not be applied to quantitative findings, as the synthesis is descriptive and does not produce pooled estimates.

Second, substantial heterogeneity is expected across interventions, settings, definitions of integration, used frameworks, types of data and reporting practices. This requires coding, theme development, mixed-methods integration and mapping to frameworks such as CFIR, which introduces subjectivity. These risks will be mitigated through independent double extraction, consensus procedures and transparent documentation of decisions. Finally, publication and reporting biases are likely, particularly because implementation processes are often poorly documented. This may also lead to under-representation of key constructs in implementation strategies and outcomes.

### Ethics and dissemination

This systematic review does not require ethical approval because it involves analysing only published data and does not involve collecting primary data from human participants. At no point will personal or identifiable information be accessed or processed during the review. The review has been registered with PROSPERO under registration number CRD420261321825 to ensure transparency and prevent duplication of research efforts. Any amendments to the protocol will be documented in PROSPERO, including the date, description, and rationale for each change.

The findings from this review will be shared through various channels to maximise reach among academic, policy and practice audiences. The completed review will be submitted for publication in a peer-reviewed open-access journal to ensure broad accessibility, particularly for audiences in LMIC health systems, where evidence on integrated care implementation is most needed. Additionally, results will be presented at relevant international conferences focused on implementation science, global health and cardiometabolic and MNS healthcare.

Since this review is part of the M-CARE project (funded by the European Commission, Grant Agreement No. 101226563), the findings will also be shared with project partners and disseminated through M-CARE’s communication channels. This will include policy briefs and practitioner-facing summaries designed to help translate evidence into actionable guidance. Where applicable, findings will be shared with WHO technical teams working on the PEN and the mhGAP to inform future guidance on integrated service delivery.

All data supporting the review, including the final search strings, PRISMA flow diagram, data extraction forms, coding frameworks and synthesis outputs, will be made available in an open-access repository on publication to promote transparency and reproducibility.

## Supplementary material

10.1136/bmjopen-2026-125828 online supplemental file 1

## Data Availability

Data are available upon reasonable request.
