# Supplementary material for: Implementation strategies, outcomes and determinants of integrated care for cardiometabolic conditions and mental, neurological and substance use disorders: protocol for a mixed-methods systematic review
Source: BMJ Open. 2026 Oct 21;16(10):e125828. doi: 10.1136/bmjopen-2026-125828 (PMC13691822; doi:10.1136/bmjopen-2026-125828)
Supplement: Supplementary file 1 [file bmjopen-2026-125828.reviewer_comments.pdf]

PEER REVIEW HISTORY

BMJ Open publishes all reviews undertaken for accepted manuscripts. Reviewers are asked to complete a checklist review form and are provided with free text boxes to elaborate on their assessment. These free text comments are reproduced below.

ARTICLE DETAILS

Title (Provisional)

Implementation Strategies, Outcomes, and Determinants of Integrated Care for Cardiometabolic conditions and Mental, Neurological and Substance use Disorders: Protocol for a Mixed-Methods Systematic Review.

Authors

Blinker, Emma Jeltje; Amoh, Gordon Kwasi Ampomah; Ankamah, Samuel; Agyemang, Charles; Omech, Bernard; Beune, Erik; Wekesah, Frederick Murunga; Azumah, Marilyn; Munneke, Wouter

VERSION 1 - REVIEW

**Reviewer**	**1**
**Name**	**BALHARA, YATAN**
**Affiliation**	**Dr.RML PGIMER AND HOSPITAL, NEW DELHI, PSYCHIATRY**
**Date**	**29-Aug-2026**
**COI**	**None**

This is a relevant and potentially useful protocol for a mixed-methods systematic review of implementation of integrated cardiometabolic and mental health care. The following are my comments on the article-

The primary question being addressed in the proposed review is “Which implementation strategies have been used … and what implementation outcomes are associated with these strategies?” However, the quantitative synthesis is explicitly limited to descriptive frequencies and proportions, with no secondary statistical analysis or effect-size pooling. Frequencies of strategies and frequencies of outcomes can establish that a strategy and an outcome were reported, but not that an implementation strategy was associated with, contributed to, or improved a particular implementation outcome. This needs to be addressed.

The eligibility criteria require integrated cardiometabolic and mental health management “within a shared care pathway” and exclude referral-only or parallel uncoordinated services.

The ‘integration” is likely to be highly heterogeneous across the included literature and hence required more precise operationlaisation.

The eligibility criteria define mental health conditions according to WHO mhGAP as including depression, anxiety, PTSD, dementia, epilepsy/seizures, psychosis, bipolar disorder, self-harm/suicide and substance-use disorders. The article uses the phrase mental, neurological and substance abuse disorders (MNS). This should be reflected in all the sections of the article including the title.

The manuscript repeatedly states that implementation outcomes will be classified using Proctor et al.'s taxonomy. The established Proctor implementation-outcomes taxonomy comprises eight outcomes: acceptability, adoption, appropriateness, feasibility, fidelity, implementation cost, penetration and sustainability. The manuscript instead lists outcomes including reach, while omitting appropriateness from several descriptions. This needs to be corrected.

The protocol proposes deductive coding of implementation strategies using ERIC. However, integrated-care studies frequently describe clinical intervention components and implementation strategies together. The protocol needs to distinguish implementation strategies from intervention components.

The PubMed strategy requires four concepts: cardiometabolic/NCD conditions; mental health conditions; integrated care; and implementation/outcomes. The eligibility criteria explicitly include studies reporting barriers, facilitators and stakeholder experiences, even when they may not use implementation-science terminology.

The use of ‘convergent segregated’ and ‘narrative synthesis’ should be clarified.

The protocol should specify how mixed-methods studies will be handled.

VERSION 1 - AUTHOR RESPONSE

Reviewer: 1
Dr. YATAN BALHARA, Dr.RML PGIMER AND HOSPITAL, NEW DELHI
Comments to the Author:
This is a relevant and potentially useful protocol for a mixed-methods systematic review of implementation of integrated cardiometabolic and mental health care. The following are my comments on the article-

RE: Thank you for carefully reading our mixed-method review protocol, highlighting the relevance and potential, but also sharing your comments to enhance the quality of the manuscript. We have aimed to address your comments carefully and have described the changes underneath each comment.

The primary question being addressed in the proposed review is “Which implementation strategies have been used … and what implementation outcomes are associated with these strategies?” However, the quantitative synthesis is explicitly

limited to descriptive frequencies and proportions, with no secondary statistical analysis or effect-size pooling. Frequencies of strategies and frequencies of outcomes can establish that a strategy and an outcome were reported, but not that an implementation strategy was associated with, contributed to, or improved a particular implementation outcome. This needs to be addressed.

RE: We fully acknowledge the limitation of no statistical analysis or effect-size pooling. Implementation outcomes are usually reported with descriptions like proportions, counts, or narrative assessments rather than statistically testes effect sizes. Therefore, we revised the research aims into narratively synthesizing the implementation strategies used to integrate care for cardiometabolic diseases and mental, neurological and substance use disorders across global settings, and their reported implementation outcomes instead of question with implementation outcomes are associated with implementation strategies. It is revised accordingly:

Therefore, the primary aim is to narratively synthesize the implementation strategies used to integrate care for cardiometabolic diseases and mental, neurological and substance use disorders across global settings, and their reported implementation outcomes.

Secondary aims are:

1) *To identify which implementation strategies are commonly applied to integrate care for cardiometabolic diseases and mental, neurological and substance use disorders.*
2) *To identify the frequency of implementation outcomes (e.g., acceptability, feasibility, fidelity, adoption, sustainability, and cost) examined in relation to integrated care.*
3) *To identify which determinants (e.g., health system capacity, workforce, cultural context, and financing) influenced successful implementation of integrated care.*
4) *To identify how do implementation strategies and reported outcomes differ between LMICs, and HICs.*

This is further addressed in the data synthesis paragraph where we stated that the synthesis will identify patterns in how integrated care models, implementation strategies and outcomes are co reported across studies and that this do not imply statistical or causal relationships:

Data synthesis

To address the review questions, the synthesis will identify patterns in how integrated-care models, implementation strategies, and outcomes are co-reported across studies. We will conduct a mixed-methods, framework-based convergent segregated synthesis following the Joanna Briggs Institute (JBI) methodology for mixed-methods systematic reviews (53–55). In this design, quantitative and qualitative data are analyzed independently, using methods appropriate to each data type, and subsequently integrated at the level of findings, followed by integration of the two sets of findings. For mixed-methods studies, quantitative and qualitative components will be extracted separately, ensuring that both contribute to the synthesis. Quantitative data will be synthesized narratively, given the heterogeneity of outcomes and the absence of effect sizes, while qualitative data will be analyzed inductively through thematic approaches. During integration of both findings, quantitative and qualitative evidence will be

given equal priority. This data synthesis identifies narrative patterns and does not imply statistical or causal relationships.

The eligibility criteria require integrated cardiometabolic and mental health management "within a shared care pathway" and exclude referral-only or parallel uncoordinated services. The 'integration" is likely to be highly heterogeneous across the included literature and hence required more precise operationlaisation.

RE: We agree that integration is likely to be highly heterogeneous across the included literature. To address this expected heterogeneity, we will extract and classify the *level of integration* as a study variable. In addition, we will produce a brief, narrative summary of each integrated-care model's key elements (e.g., non-specialist mental health treatment, case-manager supervision, electronic decision-support tools). Each model will be summarised to describe how cardiometabolic and MNS services are combined within the shared care pathway. We revised the manuscript accordingly:

Qualitative analysis

Qualitative data will comprise descriptive information about integrated care models, implementation strategies, verbatim excerpts, stakeholder narratives, case descriptions, and open-ended survey responses from included studies. These will first be analyzed inductively through open coding to identify emergent characteristics and themes. To ensure conceptual clarity between a model of care and implementation strategies, we define a model of care, in line with WHO, in line with WHO as the overarching concept for how services are delivered, including the processes of care, the organisation of providers, and the management of services [59]. The key characteristics of each integrated-care model will be synthesized and reported in a structured table.

The eligibility criteria define mental health conditions according to WHO mhGAP as including depression, anxiety, PTSD, dementia, epilepsy/seizures, psychosis, bipolar disorder, self-harm/suicide and substance-use disorders. The article uses the phrase mental, neurological and substance abuse disorders (MNS). This should be reflected in all the sections of the article including the title.

RE: Throughout the manuscript we have changed mental health conditions into mental, neurological and substance abuse disorders, including the title:

Title:

Implementation Strategies, Outcomes, and Determinants of Integrated care for Cardiometabolic conditions and Mental, Neurological and Substance use Disorders: Protocol for a Mixed-Methods Systematic Review.

The manuscript repeatedly states that implementation outcomes will be classified using Proctor et al.'s taxonomy. The established Proctor implementation-outcomes taxonomy comprises eight outcomes: acceptability, adoption, appropriateness, feasibility, fidelity, implementation cost, penetration and sustainability. The

manuscript instead lists outcomes including reach, while omitting appropriateness from several descriptions. This needs to be corrected.

RE: We have removed reach from the descriptions in the manuscript and added appropriateness to all sections.

The protocol proposes deductive coding of implementation strategies using ERIC. However, integrated-care studies frequently describe clinical intervention components and implementation strategies together. The protocol needs to distinguish implementation strategies from intervention components.

RE: To address the reported overlap in care models and implementation strategies, we have clarified our qualitative analysis to explicitly distinguish between the two:

Qualitative analysis

Qualitative data will comprise descriptive information about integrated care models, implementation strategies, verbatim excerpts, stakeholder narratives, case descriptions, and open-ended survey responses from included studies. These will first be analyzed inductively through open coding to identify emergent characteristics and themes. To ensure conceptual clarity between a model of care and implementation strategies, we define a model of care, in line with WHO, in line with WHO as the overarching concept for how services are delivered, including the processes of care, the organisation of providers, and the management of services [59]. The key characteristics of each integrated-care model will be synthesized and reported in a structured table. Implementation strategies are defined, following Proctor et al. (2013), as "methods or techniques used to enhance the adoption, implementation, and sustainability of a clinical program or practice" [60]. Each strategy will be documented with its name, conceptual definition, and operationalisation. The inductive codes of implementation strategies, outcomes and determinants will then be deductively mapped onto three distinct implementation framework components: …..

The PubMed strategy requires four concepts: cardiometabolic/NCD conditions; mental health conditions; integrated care; and implementation/outcomes. The eligibility criteria explicitly include studies reporting barriers, facilitators and stakeholder experiences, even when they may not use implementation-science terminology.

RE: Indeed, therefore the terminology related to determinants, barriers, and facilitators, are included in the search strategy. To clarify this in the manuscript, we have revised the search strategy section accordingly:

"Database searches are conducted in PubMed, CINAHL, APA PsycINFO, Scopus, and Web of Science, combining MeSH terms and free-text keywords related to cardiometabolic diseases, MNS conditions, integrated care, implementation strategies, and implementation outcomes including determinants, barriers, facilitators, and stakeholder experiences."

The use of 'convergent segregated' and 'narrative synthesis' should be clarified.

RE: Convergent segregated synthesis refers to the overall mixed-methods design, where quantitative and qualitative evidence are analysed separately and then integrated at the findings level. Narrative synthesis is the method we apply specifically to the quantitative strand, while qualitative data will be synthesised thematically. This distinction ensures clarity: the design is convergent segregated, and narrative synthesis is the analytic technique within one strand.

To address the review questions, the synthesis will identify patterns in how integrated-care models, implementation strategies, and outcomes are co-reported across studies. We will conduct a mixed-methods, framework-based convergent segregated synthesis following the Joanna Briggs Institute (JBI) methodology for mixed-methods systematic reviews (53–55). In this design, quantitative and qualitative data are analyzed independently, using methods appropriate to each data type, and subsequently integrated at the level of findings, followed by integration of the two sets of findings. For mixed-methods studies, quantitative and qualitative components will be extracted separately, ensuring that both contribute to the synthesis. Quantitative data will be synthesized narratively, given the heterogeneity of outcomes and the absence of effect sizes, while qualitative data will be analyzed inductively through thematic approaches. During integration of both findings, quantitative and qualitative evidence will be given equal priority. This data synthesis identifies narrative patterns and does not imply statistical or causal relationships.

The protocol should specify how mixed-methods studies will be handled.
Mixed-methods studies will be analysed using a convergent segregated approach. Quantitative and qualitative components will be extracted separately to preserve the integrity of each data, and synthesised in parallel (narrative for quantitative, thematic for qualitative), and then integrated at the findings level. This process is specified in the revised data synthesis section:

To address the review questions, the synthesis will identify patterns in how integrated-care models, implementation strategies, and outcomes are co-reported across studies. We will conduct a mixed-methods, framework-based convergent segregated synthesis following the Joanna Briggs Institute (JBI) methodology for mixed-methods systematic reviews (53–55). In this design, quantitative and qualitative data are analyzed independently, using methods appropriate to each data type, and subsequently integrated at the level of findings, followed by integration of the two sets of findings. For mixed-methods studies, quantitative and qualitative components will be extracted separately, ensuring that both contribute to the synthesis. Quantitative data will be synthesized narratively, given the heterogeneity of outcomes and the absence of effect sizes, while qualitative data will be analyzed inductively through thematic approaches. During integration of both findings, quantitative and qualitative evidence will be given equal priority. This data synthesis identifies narrative patterns and does not imply statistical or causal relationships.
