# Supplementary material for: Implementation strategies, outcomes and determinants of integrated care for cardiometabolic conditions and mental, neurological and substance use disorders: protocol for a mixed-methods systematic review
Source: BMJ Open. 2026 Oct 21;16(10):e125828. doi: 10.1136/bmjopen-2026-125828 (PMC13691822; doi:10.1136/bmjopen-2026-125828)
Supplement: Supplementary file 2 [file bmjopen-16-10-s001.docx]

**Supplemental material**

**Search strategy for PubMed (to be tailored for the other databases)**

| **Search number** | **Search Details** | **Results** |
| --- | --- | --- |
| #1. Non-communicable diseases and multimorbidity search terms | "Noncommunicable Diseases"[MeSH Terms] OR "Multiple Chronic Conditions"[MeSH Terms] OR "Cardiovascular Diseases"[MeSH Terms] OR "Diabetes Mellitus"[MeSH Terms] OR "Dyslipidemias"[MeSH Terms] OR "Metabolic Syndrome"[MeSH Terms] OR "Comorbidity"[MeSH Terms] OR "Hypertension"[All Fields] OR "diabetes"[All Fields] OR "Diabetes Mellitus"[All Fields] OR "dyslipidemia"[All Fields] OR "dyslipidaemia"[All Fields] OR "Dyslipidemias"[All Fields] OR "cardiovascular disease"[All Fields] OR "CVD"[All Fields] OR "CVDS"[All Fields] OR "cardiometabolic disease"[All Fields] OR "Chronic Disease"[All Fields] OR "chronic condition"[All Fields] OR "Cardiovascular Diseases"[All Fields] OR "cardiometabolic diseases"[All Fields] OR "chronic diseases"[All Fields] OR "chronic conditions"[All Fields] OR "Multiple Chronic Conditions"[All Fields] OR "multiple long-term conditions"[All Fields] OR "multiple long term complications"[All Fields] OR "multiple long-term conditions"[All Fields] OR "noncommunicable disease"[All Fields] OR "Noncommunicable Diseases"[All Fields] OR "non-communicable disease"[All Fields] OR "non-communicable diseases"[All Fields] OR "NCD"[All Fields] OR "NCDs"[All Fields] OR "Comorbid"[All Fields] OR "co-morbid"[All Fields] OR "co-morbidity"[All Fields] OR "comorbidities"[All Fields] OR "co-morbidities"[All Fields] OR "Comorbidity"[All Fields] OR "multi-morbid"[All Fields] OR "multimorbidity"[All Fields] OR "multiple condition"[All Fields] OR "multi-condition"[All Fields] OR "multi-condition"[All Fields] OR "co-existing condition"[All Fields] OR "coexisting condition"[All Fields] OR "co-occurring condition"[All Fields] OR "co-occurring conditions"[All Fields] OR "cooccurring conditions"[All Fields] OR "elevated blood pressure"[All Fields] OR "high blood pressure"[All Fields] OR "raised blood pressure"[All Fields] OR "arterial blood pressure"[All Fields] OR "diabetes mellitus type 2"[All Fields] OR "diabetes type 2"[All Fields] OR "T2DM"[All Fields] OR "diabetes type II"[All Fields] OR "Stroke"[All Fields] OR "cerebrovascular disease"[All Fields] OR "cerebrovascular accident"[All Fields] OR "brain ischemia"[All Fields] OR "cerebral infarction"[All Fields] OR "ischemic heart disease"[All Fields] OR "coronary heart disease"[All Fields] OR "peripheral vascular diseases"[All Fields] OR "heart disease"[All Fields] OR "cardiac disease"[All Fields] OR "ischemic heart diseases"[All Fields] OR "coronary artery disease"[All Fields] OR "heart failure"[All Fields] OR "peripheral arterial disease"[All Fields] OR "heart diseases"[All Fields] OR "Hypercholesterolemia"[All Fields] OR "hypercholesterolaemia"[All Fields] OR "high cholesterol"[All Fields] OR "elevated cholesterol"[All Fields] OR "raised cholesterol"[All Fields] OR "hyperlipidemia"[All Fields] OR "hyperlipidemias"[All Fields] OR "hyperlipidaemia"[All Fields] OR "hyperlipidaemias"[All Fields] OR "Metabolic Diseases"[All Fields] OR "Metabolic Syndrome"[All Fields] OR "who-pen"[All Fields] OR "who-pen"[All Fields] | 5,067,871 |
| #2. Mental, neurological and substance use disorder search terms | "Mental Disorders"[MeSH Terms] OR "Anxiety"[MeSH Terms] OR "Epilepsy"[MeSH Terms] OR "Epilepsy"[All Fields] OR "Seizures"[MeSH Terms] OR "Depression"[All Fields] OR "Depressive Disorder"[All Fields] OR "Anxiety"[All Fields] OR "mental health condition"[All Fields] OR "mental health conditions"[All Fields] OR "mental illness"[All Fields] OR "mental illnesses"[All Fields] OR "Mental Disorders"[All Fields] OR "Mental Disorder"[All Fields] OR "substance use"[All Fields] OR "alcohol use"[All Fields] OR "alcohol abuse"[All Fields] OR "alcohol misuse"[All Fields] OR "alcoholism"[All Fields] OR "alcohol addiction"[All Fields] OR "Substance-Related Disorders"[All Fields] OR "Substance-Related Disorder"[All Fields] OR "Alcohol-Related Disorders"[All Fields] OR "Alcohol-Related Disorder"[All Fields] OR "drug use"[All Fields] OR "drug abuse"[All Fields] OR "drug misuse"[All Fields] OR "drug addiction"[All Fields] OR "psychiatric disorder"[All Fields] OR "affective disorder"[All Fields] OR "affective disorders"[All Fields] OR "Psychotic Disorders"[All Fields] OR "Psychotic Disorder"[All Fields] OR "Bipolar Disorder"[All Fields] OR "Self-Injurious Behavior"[MeSH Terms] OR "Self-Injurious Behavior"[All Fields] OR "self-harm"[All Fields] OR "self-harm"[All Fields] OR "suicide"[All Fields] OR "suicidal"[All Fields] OR "Schizophrenia"[All Fields] OR "hypomania"[All Fields] OR "mania"[All Fields] OR "posttraumatic stress"[All Fields] OR "Post-traumatic stress"[All Fields] OR "stress-related mental disorders"[All Fields] OR "stress-related mental disorder"[All Fields] OR "stress-related disorders"[All Fields] OR "stress-related disorder"[All Fields] OR "depressive"[All Fields] OR "psychological disorder"[All Fields] OR "neuropsychiatric disorders"[All Fields] OR "neuropsychiatric disorder"[All Fields] OR "Psychosis"[All Fields] OR "Post-traumatic stress disorder"[All Fields] OR "panic disorder"[All Fields] OR "mhGAP"[All Fields] OR "mhGAP-ig"[All Fields] OR "mental health gap action programme"[All Fields] OR "neurodegenerative disorders"[All Fields] OR "neurodegenerative diseases"[All Fields] OR "neurodegenerative disorder"[All Fields] OR "neurodegenerative disease"[All Fields] OR "Dementia"[All Fields] OR "Alzheimer"[All Fields] OR "Neurocognitive Disorders"[All Fields] OR "Seizures"[All Fields] | 2,849,558 |
| #3. Integrated care search terms | "delivery of health care, integrated"[MeSH Terms] OR "Patient Care Team"[MeSH Terms] OR "Case Management"[MeSH Terms] OR "care integration"[All Fields] OR "integrated care"[All Fields] OR "integrated chronic care"[All Fields] OR "integrated healthcare"[All Fields] OR "integrated health care"[All Fields] OR "integrated primary health care"[All Fields] OR "integrated primary healthcare"[All Fields] OR "integrated health systems"[All Fields] OR "integrated health system"[All Fields] OR "integrated systems"[All Fields] OR "integrated system"[All Fields] OR "integrated models"[All Fields] OR "integrated model"[All Fields] OR "integrated approach"[All Fields] OR "integrated disease management"[All Fields] OR "integrated delivery system"[All Fields] OR "integrated service delivery"[All Fields] OR "integrated health services"[All Fields] OR "integrated patient-centred care"[All Fields] OR "integrated people-centred health services"[All Fields] OR "integrated community case management"[All Fields] OR "ICMM"[All Fields] OR "collaborative care"[All Fields] OR "collaborative health care"[All Fields] OR "collaborative healthcare"[All Fields] OR "coordinated care"[All Fields] OR "coordinated health care"[All Fields] OR "coordinated healthcare"[All Fields] OR "team-based care"[All Fields] OR "multidisciplinary care"[All Fields] OR "interdisciplinary care"[All Fields] OR "interdisciplinary approach"[All Fields] OR "interdisciplinary model"[All Fields] OR "interdisciplinary collaboration"[All Fields] OR "inter-disciplinary care"[All Fields] OR "cross-disciplinary care"[All Fields] OR "cross-disciplinary approach"[All Fields] OR "cross-disciplinary collaboration"[All Fields] OR "cross sectoral care"[All Fields] OR "transdisciplinary approach"[All Fields] OR "transdisciplinary model"[All Fields] OR "transdisciplinary care"[All Fields] OR "biopsychosocial care"[All Fields] OR "biopsychosocial treatment"[All Fields] OR "biopsychosocial approach"[All Fields] OR "biopsychosocial management"[All Fields] OR "biopsychosocial model"[All Fields] OR "patient care management"[All Fields] OR "patient-centred care"[All Fields] OR "patient-centered care"[All Fields] OR "person-centred care"[All Fields] OR "person-centered care"[All Fields] OR "people-centred care"[All Fields] OR "people-centered care"[All Fields] OR "task shifting"[All Fields] OR "task sharing"[All Fields] OR "stepped care"[All Fields] OR "service integration"[All Fields] OR "shared care"[All Fields] OR "care pathway"[All Fields] OR "care pathways"[All Fields] OR "care model"[All Fields] OR "care models"[All Fields] OR "integration of care"[All Fields] OR "integration of services"[All Fields] OR "Integrated health"[All Fields] OR "comprehensive care"[All Fields] OR "comprehensive healthcare"[All Fields] OR "interprofessional care"[All Fields] OR "clinical pathway"[All Fields] OR "clinical integration"[All Fields] OR "delivery of health care"[All Fields] OR "continuity of care"[All Fields] OR "delivery of health care integrated"[All Fields] OR "co-location"[All Fields] OR "one stop shop"[All Fields] OR "one-stop care"[All Fields] | 399,785 |
| # 4. Implementation outcomes including determinants, barriers, facilitators and stakeholder experiences. | "Implementation Science"[MeSH Terms] OR "Program Evaluation"[MeSH Terms] OR "process assessment, health care"[MeSH Terms] OR "Health Services Accessibility"[MeSH Terms] OR "Patient Acceptance of Health Care"[MeSH Terms] OR "Costs and Cost Analysis"[MeSH Terms] OR "Health Planning"[MeSH Terms] OR "acceptability"[All Fields] OR "adoption"[All Fields] OR "appropriateness"[All Fields] OR "feasibility"[All Fields] OR "fidelity"[All Fields] OR "penetration"[All Fields] OR "reach"[All Fields] OR "sustainability"[All Fields] OR "uptake"[All Fields] OR "adherence"[All Fields] OR "implementation outcome"[All Fields] OR "implementation outcomes"[All Fields] OR "barriers"[All Fields] OR "facilitators"[All Fields] OR "contextual factors"[All Fields] OR "health system factors"[All Fields] OR "implementation cost"[All Fields] OR "intervention cost"[All Fields] OR "program cost"[All Fields] OR "cost"[All Fields] OR "costs"[All Fields] OR "cost-effectiveness"[All Fields] OR "cost-benefit"[All Fields] OR "economic"[All Fields] OR "continuation"[All Fields] OR "maintenance"[All Fields] OR "implementation research"[All Fields] OR "process evaluation"[All Fields] OR "Implementation determinants"[All Fields] OR "determinants of implementation"[All Fields] OR "determinants of care"[All Fields] OR "Implementation Science"[All Fields] OR "Program Evaluation"[All Fields] OR "hybrid effectiveness-implementation"[All Fields] OR "effectiveness-implementation hybrid"[All Fields] OR "scalability"[All Fields] OR "scale-up"[All Fields] OR "coverage"[All Fields] OR "barrier"[All Fields] OR "facilitator"[All Fields] OR "RE-AIM"[All Fields] OR "CFIR"[All Fields] OR "PARiHS"[All Fields] | 4,400,718 |
| # 5 | #1 AND #2 AND #3 AND #4 | 4,473 |
